# Short-Term Changes in Arterial Stiffness Measured by 2D Speckle Tracking in Patients Undergoing Transcatheter Aortic Valve Implantation

**DOI:** 10.3390/jcm13010222

**Published:** 2023-12-30

**Authors:** Leonie Arnold, Nikolaus Alexander Haas, André Jakob, Julius Fischer, Steffen Massberg, Simon Deseive, Felix Sebastian Oberhoffer

**Affiliations:** 1 Division of Pediatric Cardiology and Intensive Care, University Hospital, LMU Munich, 81377 Munich, Germany; 2 Department of Medicine I, University Hospital, LMU Munich, 81377 Munich, Germany

**Keywords:** transcatheter aortic valve replacement, vascular stiffness, 2D speckle tracking

## Abstract

Arterial stiffness has received increasing interest as a cardiovascular marker in patients with aortic valve stenosis (AS). So far, studies on the impact of aortic valve replacement (AVR) on arterial stiffness have been equivocal. Two-dimensional speckle tracking (2DST) is a novel, non-invasive method to measure the motion of the vessel wall. In this prospective observational study, we aimed to assess the change in arterial stiffness of the common carotid artery (CCA) measured by 2DST in patients undergoing transcatheter aortic valve implantation (TAVI). A total of 47 patients were included in the study (age 80.04 ± 6.065 years). Peak circumferential strain (CS) was significantly improved after TAVI (4.50 ± 2.292 vs. 5.12 ± 2.958, *p* = 0.012), as was the peak strain rate (CSR) (0.85 ± 0.567 vs. 1.35 ± 0.710, *p* = 0.002). Body mass index (BMI), mean arterial pressure (MAP) and hemodynamic parameters were associated with this change. 2DST results did not correlate with aortic pulse wave velocity (aPWV) or augmentation index normalized to heart rate (AIx@75), suggesting a distinct difference between arterial stiffness of the CCA and other stiffness parameters. 2DST seems to be a promising new tool to assess arterial stiffness in TAVI patients.

## 1. Introduction

Aortic valve stenosis (AS) is the most common acquired valvular heart disease. As the prevalence increases with age, the burden of disease is only expected to rise in the future [1]. Mortality is low in patients with asymptomatic AS. However, once symptoms occur, the mortality rate increases dramatically unless aortic valve replacement (AVR) is performed [2]. Transcatheter aortic valve implantation (TAVI) is now more common in Germany than surgical valve replacement, and there is ongoing investigation into its indication in different low-risk groups [3,4].

In the general population, arterial stiffness has been established as an independent marker and predictor of cardiovascular events and all-cause mortality [5,6]. Interestingly, arterial stiffness is also gaining more and more interest as a prognostic cardiovascular marker in AS patients [7].

In recent years, a better understanding of the changes in central aortic stiffness after AVR has emerged. Central pulse wave velocity (PWV) increases as an immediate adaption to the load change after the reparation of the damaged valve. Studies suggest that this adaptation is not only limited to the aorta but also applies to other parts of the vascular system [8,9]. However, relevant data on this matter are scarce.

Two-dimensional speckle tracking (2DST) is a novel, non-invasive imaging technique to measure arterial stiffness of the common carotid artery (CCA). 2DST has wide application in the assessment of left ventricular function. It has also been proposed as a direct measure to evaluate arterial stiffness of vessels, such as the CCA [10]. It can be used as a screening tool during routine sonographic examination and is easy to apply.

The objectives of this study were to assess the change in arterial stiffness of the CCA in patients undergoing TAVI using 2DST and to compare these results to other arterial stiffness parameters. Moreover, to investigate associations between arterial stiffness, its change after TAVI, and patient characteristics.

## 2. Materials and Methods

We conducted this study in accordance with the revised version of the Declaration of Helsinki [11]. The local ethics committee “Ethikkommission der Medizinischen Fakultät der LMU München“ approved this study (project number: 21-0418, date of approval: 1 June 2021). All patients gave written informed consent. This prospective observational study took place between August 2021 and March 2022. Patients were recruited at the Department of Medicine I, University Hospital, LMU Munich. All patients scheduled for TAVI procedure that met the inclusion criteria were contacted for participation. Inclusion criteria were severe AS and a referral for TAVI. Severe AS was defined according to the guidelines of the joint taskforce of the European Society of Cardiology and the European Association for Cardio-Thoracic Surgery, and the German extension of the guidelines [12,13]. General exclusion criteria were peripheral artery and/or neurological disease and a history of carotid endarterectomy. Only patients who completed pre- and post-TAVI measurements were included in the study sample.

Patients were examined 24 to 48 h prior to TAVI and 72 h after TAVI. If patients suffered from TAVI-associated complications, this period was extended until the second examination was possible. Patients who could not be examined 14 days after TAVI were excluded. All patients were examined by the same study investigator. Information on patients’ medical history, including pre-existing health conditions (arterial hypertension, diabetes, atrial fibrillation, coronary artery disease, lipid metabolism disorders, chronic renal disease), NYHA class, smoking status, regular medication (coumarin, acetylsalicylic acid, clopidogrel, beta-blockers, angiotensin-converting enzyme inhibitors, angiotensin receptor blockers, diuretics, statins), and laboratory work (NT-proBNP, total cholesterol, triglycerides, LDL-cholesterol, HDL-cholesterol, non-HDL-cholesterol) were taken from clinical records and by questioning patients. Information on peak aortic valve velocity (PVel, m/s), mean pressure gradient (MPG, mmHg), maximum pressure gradient (MaxPG, mmHg), and aortic valve area (AVA, mm^2^) were evaluated retrospectively through routine transthoracic echocardiography as part of the conventional TAVI examination.

**Figure 1:**
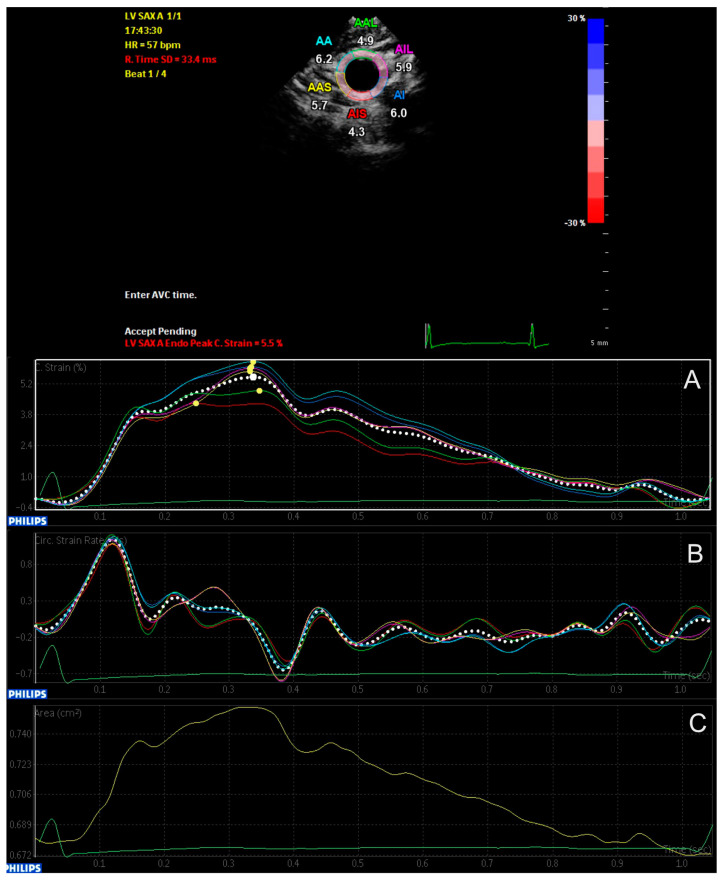
Two−dimensional speckle (2DST) tracking of the common carotid artery (CCA) for the measurement of (**A**) the region of interest and the peak circumferential strain (CS), (**B**) the peak circumferential strain rate (CSR) and (**C**) the change of area.

Sonographic examination of the CCA was performed by one investigator using a 3–8 MHz sector array transducer on a Philips iE33 xMatrix ultrasound machine (Philips Healthcare, Amsterdam, Netherlands). Loops were recorded under constant three-lead ECG. After 15 minutes of resting, patients were examined in a supine position with the neck extended at a 45° degree angle facing away from the investigator. Patients were instructed to hold their breath and not swallow during recordings, to minimize motion artifacts. Bilateral B-Mode sonographic recordings were taken approximately 1 cm below the carotid bifurcation over three consecutive heart cycles. The recordings were transferred to a workstation for further offline analysis (QLAB cardiovascular ultrasound quantification software version 11.1, Philips Healthcare, Amsterdam, Netherlands). Offline analysis was performed on sonographic recordings that met sufficient image quality requirements. Loops with motion artifacts were excluded. The evaluation was performed as previously described [14]. Peak circumferential strain (CS, %), the maximal deformation of the vessel wall in percent, and peak strain rate (CSR, 1/s), the maximal change of circumferential strain over time, were calculated semi-automatically. The software’s SAX-A function and aCMQ tool were utilized. Loops of the left and right CCA were analyzed individually. The region of interest (ROI) was manually set to exactly match the endovascular border. The software then automatically tracked the movement of the speckles within the ROI (Figure 1). The procedure was repeated three times, and the resulting values for CS, CSR, and the change in the vessel’s change in area were averaged.

In addition, the stiffness index β (β_area_) was calculated from sonographic data of the CCA. As proposed by Cho and Kim, the vessel’s area, instead of diameter, was utilized [15]. The area offers more precise information regarding the vessel’s deformation in comparison to the circumferential and longitudinal diameters. As the stiffness index β is dependent on blood pressure, which can be impacted by AVR, ẞ_area_ was normalized to blood pressure. For this reason, the reference blood pressure BP_ref_ was set to 100 mmHg, as proposed by Spronck et al. [16]:

$$\beta_{\mathrm{area}}=\frac{\ln\left(\mathrm{SBP}/\mathrm{DBP}\right)}{\left({\mathrm{area}}_{\max}/{\mathrm{area}}_{\min}\right)-1}-\ln\left(\frac{\mathrm{DBP}}{{\mathrm{BP}}_{\mathrm{ref}}}\right).$$

Blood pressure was taken from the brachial oscillometric measurement instead of local CCA pressure measurements, as suggested by some authors [17]. We deemed the necessary consistency to be provided, and the method to be sufficiently accurate due to the paired nature of the data.

Systolic (SBP, mmHg) and diastolic blood pressure (DBP, mmHg), mean arterial pressure (MAP, mmHg), aortic PWV (aPWV, m/s), augmentation index normalized to heart rate (AIx@75, %), cardiac index (CI, L/min × L/m^2^), and total vascular resistance (dyn·s/cm^5^) were measured using a non-invasive oscillometric blood pressure device with a brachial cuff and patented software with the ARCSOlver algorithm (Mobil-O-Graph, HMS CS Version 6.1, IEM GmbH, Stolberg, Germany) [18]. The Mobil-O-Graph is a validated method to measure aPWV [19]. The pulse wave is recorded at the A. brachialis over 10 s using a high-fidelity pressure sensor. A three-level algorithm analyzes the pressure waves. The algorithm tests plausibility, removes artifacts, and applies a transfer function [20]. Measurements were taken on the right arm, unless not possible. After a ten-minute acclimatization period in the supine position, measurements were repeated at least three times and averaged. The signal quality provided by the device had to be at least excellent or good, otherwise measurements were discarded. 

We conducted a sample size calculation prior to this study. A mean of 4.0% and a standard deviation (SD) of 1.0% were estimated for CS from the literature. A 10% post-TAVI change was assumed, as no literature is available on the change in CS for patients post-TAVI. Adding a dropout rate of 20%, the necessary number of recruited patients was estimated to be 60.

Data were visually inspected and tested for normal distribution using qq-plots, histograms, and the Shapiro–Wilk test. Depending on the distribution, patients’ characteristics were presented as mean ± SD, median ± IQR (interquartile range), or the number of patients (n) and percentage. Pre- and post-TAVI values of hemodynamic parameters and stiffness indices were compared by paired t-tests or Wilcoxon rank sum tests. Additionally, an adjusted *p*-value was produced by linear mixed regression with a random intercept for patient ID and the covariates age, sex, MAP, and heart rate (HR, bpm). HR and MAP were modeled as time-variant fixed effects, sex and age were time-invariant. β_area_ was not adjusted for MAP since the stiffness index itself is already corrected for blood pressure. Additionally, we compared the stiffness parameters of patients with atrial fibrillation (paroxysmal and permanent) to patients without atrial fibrillation by using the Wilcoxon rank sum test. To evaluate correlations between stiffness parameters, as well as factors influencing the stiffness indices, Pearson, Spearman or point-biserial correlation coefficients were calculated based on the distribution and the type of data. To assess the change, the delta was formed from the pre- and post-TAVI measurements for each patient. Covariates included sex, age, BMI, HR, MAP, hemodynamic measurements, and laboratory parameters. *p*-values < 0.05 were considered statistically significant. All analyses were performed in R version 4.2.1 (R Core Team, Vienna, Austria, 2022).

## 3. Results

### 3.1. Study Sample

Overall, 61 patients who met the inclusion criteria were recruited into the study. Of those, 14 patients were lost to follow-up or could not be examined post-TAVI and were therefore excluded. Reasons for this were: rescheduled procedure (*n* = 5), refusal of second examination/second examination not possible (*n* = 6), and patients diseased before the procedure (*n* = 3). In total, pre- and post-TAVI measurements were available for 47 patients.

The mean age of the patients was 80.04 years (±6.065) and patients were predominantly male (76.6%). A detailed illustration of patients‘ characteristics can be found in Table 1. Baseline AVA was 0.74 mm^2^, PVel changed from 3.90 m/s pre-TAVI to 2.20 m/s post-TAVI (*p* < 0.001). MPG changed from 39.16 mmHg to 11.11 mmHg post-TAVI (*p* < 0.001) (Table 2).

### 3.2. Changes in Arterial Stiffness after TAVI

**Figure 2:**
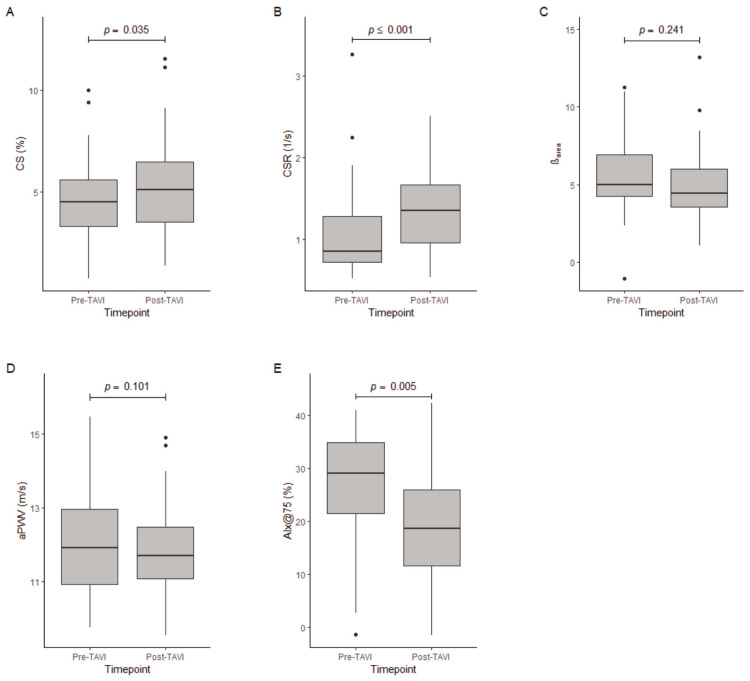
(**A**–**E**) Pre- and post-TAVI values of (**A**) CS, (**B**) CSR, (**C**) ß_area_, (**D**) aPWV, and (**E**) AIx@75.

Table 2 and Figure 2 present the changes in hemodynamic parameters and stiffness indices between pre- and post-TAVI time points. Models for the adjusted *p*-values can be found in Supplementary Table S2. CS and CSR measured by 2DST significantly increased after TAVI (CS *p* = 0.012, CSR *p* = 0.002), while the stiffness index β_area_ decreased (*p* = 0.181). aPWV decreased without reaching significance, whilst AIx@75 showed a significant decrease (aPWV *p* = 0.894, AIx@75 *p* = 0.002).

Univariate analysis revealed that a higher increase in CS was associated with a stronger decrease in MAP post-TAVI, as well as a stronger decrease in MaxPG. Patients with a lower baseline BMI and a lower baseline AVA displayed a higher increase in CS, whilst a higher baseline NT-proBNP was associated with a higher change in CS post-TAVI (Supplementary Table S1). Similar associations were found for the change in CSR post-TAVI: whilst PVel and MPG were both negatively associated with CSR, no significant influence on AVA and MAP was found. The decrease in AIx@75 was positively associated with post-TAVI HR, total peripheral resistance, as well as the change in MPG and MaxPG (Supplementary Table S1).

Due to a high rate of atrial fibrillation in the patient cohort, additional analysis comparing patients with and without atrial fibrillation was performed. No significant differences in stiffness parameters between these groups were revealed (Supplementary Table S3).

### 3.3. Assessment of Arterial Stiffness

Pre-TAVI CS was negatively associated with age, as well as pre- and post-TAVI HR, whilst post-TAVI CS was no longer associated with age and pre-TAVI HR. Only post-TAVI HR remained as a significant, yet attenuated, correlation. In comparison, CSR was neither associated with age, nor HR, but negatively correlated with ΔMPG (Supplementary Table S1).

Pre- and post-TAVI β_area_ showed a similar picture. There were no correlations with baseline patient characteristics or hemodynamic measurements. Pre-TAVI aPWV was positively associated with age and NT-proBNP, whilst post-TAVI aPWV was also associated with post-TAVI HR. Pre- and post-TAVI AIx@75 were influenced by CI and total vascular resistance (Supplementary Table S1).

### 3.4. Agreement between Stiffness Parameters

CS and CSR did not correlate with aPWV before or after TAVI, or when looking at the change of these parameters. Only pre-TAVI CS and pre-TAVI aPWV showed a moderate significant correlation (Table 3). Correlations between CS, CSR, and AIx@75 were even lower, with no significant relationships at any time point. CS correlated slightly better with β_area_ than CSR, but all the time points and the change showed significant correlations for both stiffness parameters (Table 3). Pre-TAVI CS and β_area_ and post-TAVI CS and β_area_ had the highest agreement (pre-TAVI r = −0.71, *p* < 0.001; post-TAVI r = −0.80, *p* < 0.001) and pre-TAVI CSR and β_area_ and ΔCSR and β_area_ the lowest (pre-TAVI r = −0.67, *p* < 0.001; Δpre- and post-TAVI r = −0.34, *p* < 0.001).

## 4. Discussion

Our study showed a significant improvement in the arterial stiffness of the CCA as measured by 2DST, apparent by a rise in CS and CSR. Possible relationships between cardiovascular risk factors such as BMI, hemodynamic parameters such as MPG and AVA, and the change in arterial stiffness from pre- to post-TAVI were found. This extends recent discoveries about changes in central arterial stiffness and hemodynamic parameters in patients with AS undergoing AVR. It also adds to our understanding of arterial stiffness in AS patients and could be a valuable additional diagnostic and prognostic marker in the future.

Several factors most likely explain the post-TAVI decrease in arterial stiffness measured by 2DST. Whilst several recent studies have found a consistent increase in aortic stiffness measured by the gold standard carotid-femoral PWV, as well as brachial-ankle PWV and cardio-ankle vascular index, there is little understanding of arterial stiffness in other parts of the arterial tree [8,9,21,22]. Terentes-Pritzios et al. established the model of “acute load-mediated changes in elastic properties” for the aorta after AVR, and proposed an extension of this model to the peripheral vascular system [8]. In theory, peripheral vasodilation might occur to accommodate the increased stroke volume after AVR, but, so far, no study has measured this directly. The increase in CS and CRS observed in our study supports this model, offering a link between increased aortic stiffness and decreased peripheral arterial stiffness by examining the stiffness of the CCA. This takes into account that elastic properties are heterogeneous along the arterial tree and that the CCA might adapt differently after AVR than the aorta [17]. The instant change in arterial stiffness after TAVI is most likely driven by cellular elements of the vessel (e.g., mechanical properties, paracrine mediators), which facilitate short-term adjustments to the environment [23]. Whether long-term structural changes, such as an increase in collagen and elastin fibers, also occur remains unclear [8].

In our study, patients with a smaller baseline AVA, higher improvement in MPG and MaxPG post-TAVI, and a lower BMI, showed more improvement in arterial stiffness post-TAVI. This seems plausible, as patients with more severe AS might benefit more from the restoration of the normal hemodynamic flow patterns. This is in agreement with recently published results on carotid stiffness in TAVI patients [24]. Other studies also found that echocardiographic indices including AVA, MPG, and ejection time, as well as patient characteristics like age, BMI, and HR, were the most commonly reported predictors of change in arterial stiffness after TAVI [9,25,26]. Interestingly enough, age did not significantly correlate with change in arterial stiffness, as reported by other authors [9,25]. Overall, this indicates that both hemodynamic factors and CV risk factors might influence changes in arterial stiffness in our study.

Carotid 2DST is a promising new method that is still being investigated in the evaluation of arterial stiffness. Animal sheep models and in-vitro validation had good agreement with reference strain values [27,28]. Literature reference values for CS and CSR are not widely available. So far, in a patient group slightly younger than our study sample, the picture is inhomogeneous with both higher, lower, and similar ranges in CS and CSR [24,29,30,31]. This wide range of values might be explained by age, comorbidities, and generally high heterogeneity in the patient populations of the studies. CS and CSR values found in this study seem to be comparable to what is reported in the literature, but better data on reference values and their dependence on methods are needed. A comparison of carotid 2DST with PWV and other sonographic markers of arterial stiffness, like intima media thickness and AIx@75, showed a low correlation between 2DST and other markers, but good inter- and intrarater agreement [10]. We observed the same low correlation between 2DST and other markers in our study, especially with the Mobil-O-Graph measurements. AIx@75 and aPWV are markers of central arterial stiffness, and quantify arterial stiffness in a different location of the arterial tree [17]. Correlations between β_area_ and CS and CSR were notably higher. This was to be expected, as the β stiffness index is another local arterial stiffness parameter and was measured at the same location. It is interesting that only CS and CSR significantly changed after TAVI and β_area_ did not. Podgórski et al. compared 2DST of the CCA and other sonographic markers to PWV and the augmentation index [10]. They found the reliability of 2DST to be higher than that of the β stiffness index, which might also explain the different outcomes in our studies. In a larger sample, the β stiffness index might significantly change after TAVI as well.

Our study shows the following strengths: the variation of the primary outcome variables is limited by the pre- and post-TAVI measurement design. Moreover, the performance of all measurements was conducted by one investigator. Confounders like MAP, HR, age, and sex were accounted for by the adjustment in the models. Still, certain limitations should be acknowledged.

The study sample included 47 patients, but measurements were not always available for all patients due to technical issues and measurement quality. Excluding measurements that did not meet pre-defined criteria was important to achieve high data quality. Studies in larger populations are needed, especially to establish more complex interactions between factors influencing arterial stiffness. It was not possible to blind the investigator to the time point of the measurement. The follow-up period was limited to three days, so long-term data on stiffness parameters are not available for this study. Interestingly, other studies have shown that changes in different arterial stiffness parameters persist after AVR in long-term follow-up [8,32]. However, more studies are needed to determine whether a decrease in arterial stiffness post-TAVI is associated with a better cardiovascular outcome in the long run. Further, the influence of different AVR procedures (e.g., TAVI vs. surgical valve replacement) on arterial stiffness needs to be addressed in the future. The study sample was quite heterogeneous. Men comprised the vast majority of study participants, which could be due to retention bias, as men seemed to agree more frequently to take part in the study. In addition, a larger cohort of men present for TAVI at the hospital, as studies have shown that women are underdiagnosed and the severity of AS symptoms is underestimated in female patients [33]. As expected, patients were also multimorbid. In comparison to patients receiving surgical valve replacement, TAVI patients are commonly older and frailer. This limitation was, however, accounted for by the paired study design. To minimize the influence of atrial fibrillation on the analysis, sufficient data quality in ECG-readings for 2DST had to be reached. Stiffness parameters for patients with and without atrial fibrillation were compared, and no significant differences were found. Atrial fibrillation is a common condition affecting approximately one third of TAVI patients, it is therefore important to take this factor into account [34].

## 5. Conclusions

In summary, we found a significant improvement in arterial stiffness of the CCA after TAVI, as measured by CS and CSR using 2DST, which is an accessible and readily available method to measure arterial stiffness. Results indicated that these improvements might be associated with a change in MAP, baseline AVA, BMI, ΔMPG, ΔMaxPG, and NT-proBNP for CS and CSR. Future efforts should focus on expanding the understanding of arterial stiffness in different sections of the arterial tree. Moreover, the interchangeability of 2DST with other stiffness parameters, and its additional value for prognostics, risk stratification and treatment decisions, should be addressed. Studies in larger populations and longer follow-up periods are needed to assess whether 2DST of the CCA has prognostic and diagnostic value. Additionally, more complex models investigating the factors influencing the change in both arterial stiffness of the CCA and central arterial stiffness after AVR, as well as the characteristics of patients displaying different degrees of agreement, would be beneficial to identify patients that would most benefit from AS treatment.

## Figures and Tables

**Table 1:** Baseline patients’ characteristics of the study population.

	*n*	Mean ± SD or No. (%)
**Patients’ characteristics**		
Sex (male)	47	36 (76.6%)
Age (years)	47	80.04 ± 6.065
BMI (kg/m^2^)	47	28.73 ± 4.372
Arterial hypertension	47	44 (93.6%)
Diabetes	47	15 (31.9%)
Atrial fibrillation	47	18 (38.3%)
CAD	47	27 (57.4%)
Lipid metabolism disorders	47	29 (61.7%)
Chronic renal disease	47	9 (19.1%)
Smoker (active or past)	47	15 (31.9%)
NYHA class	46	
I		6 (13.0%)
II		13 (28.3%)
III		27 (58.7%)
IV		0 (0.0%)
Time between pre-TAVI examination and TAVI procedure (hours)	47	52 ± 47.3
Time between post-TAVI examination and TAVI procedure (hours)	47	82 ± 20.5
**Medication**		
Coumarin	47	5 (10.6%)
Acetylsalicylic acid	47	27 (57.4%)
Clopidogrel	47	7 (14.9%)
Beta-blocker	47	25 (53.2%)
Angiotensin-converting enzyme inhibitor	47	19 (40.4%)
Angiotensin receptor blocker	47	12 (25.5%)
Diuretic	47	28 (59.6%)
Statin	47	18 (38.3%)
**Hemodynamic parameters**		
AVA (mm^2^)	46	0.74 ± 0.150
MaxPG (mmHg)	47	64.01 ± 17.850
MPG (mmHg)	47	39.16 ± 11.430
PVel (m/s)	46	3.90 ± 0.560
Low-flow low-gradient AS	47	8 (17.0%)
**Laboratory parameters**		
NT-proBNP (pg/mL)	45	3715.20 ± 5450.591
Total cholesterol (mg/dL)	43	177.12 ± 53.367
Triglycerides (mg/dL)	43	147.58 ± 133.655
LDL-Cholesterol (mg/dL)	43	99.05 ± 46.742
HDL-Cholesterol (mg/dL)	43	58.79 ± 17.614
Non-HDL-Cholesterol (mg/dL)	43	118.33 ± 52.251

BMI = body mass index (kg/m^2^), CAD = coronary artery disease, SD = standard deviation, AVA = aortic valve area, MPG = mean pressure gradient, MaxPG = maximum pressure gradient, PVel = peak aortic valve velocity.

**Table 2:** Change in stiffness indices and hemodynamic parameters of the patient population with aortic valve stenosis (AS) before and after transcatheter aortic valve implantation (TAVI).

		Pre-TAVI		Post-TAVI		
Parameter	N	Mean ± SD or Median ± IQR	N	Mean ± SD or Median ± IQR	*p*-Value ^1^	*p*-Value ^2^
**Stiffness indices**						
CS (%)	44	4.50 ± 2.292	43	5.12 ± 2.958	0.035	0.012
CSR (1/s)	44	0.85 ± 0.567	43	1.35 ± 0.710	<0.001	0.002
β_area_	43	4.99 ± 2.720	42	4.44 ± 2.440	0.241	0.143
aPWV (m/s)	38	11.92 ± 2.050	41	11.70 ± 1.400	0.101	0.894
AIx@75 (%)	41	29.00 ± 13.417	38	18.67 ± 14.333	0.005	0.002
**Hemodynamic parameters**						
PVel (m/s)	46	3.90 ± 0.560	45	2.20 ± 0.370	<0.001	
MPG (mmHg)	47	39.16 ± 11.430	41	11.11 ± 3.916	<0.001	
MaxPG (mmHg)	47	64.01 ± 17.850	47	19.89 ± 6.818	<0.001	
SBP (mmHg)	45	130.33 ± 18.073	45	125.60 ± 16.694	0.232	
DBP (mmHg)	45	77.92 ± 8.831	45	75.35 ± 12.274	0.239	
MAP (mmHg)	45	100.79 ± 11.137	45	96.50 ± 11.473	0.070	
CI (L/min × L/m^2^)	38	2.40 ± 0.432	41	2.60 ± 0.400	0.004	
Total vascular resistance (dyn·s/cm^5^)	38	1732.64 ± 340.212	41	1539.50 ± 222.133	0.010	
HR (bpm)	45	66.79 ± 12.275	44	72.23 ± 10.581	0.002	

^1^ *p*-value calculated by *t*-test for normal data and Wilcoxon rank sum test for non-normal data; ^2^ *p*-value derived from mixed model corrected for age, sex, heart rate, and MAP; CS = circumferential strain, CSR = circumferential strain rate, β_area_ = normalized stiffness index based on area, aPWV = arterial pulse wave velocity, AIx@75 = augmentation index normalized to heart rate.

**Table 3:** Correlation of arterial stiffness markers from 2D speckle tracking (2DST) of the common carotid artery (CCA) and aPWV, AIx@75, and β_area_ in patients with aortic valve stenosis (AS) before and after transcatheter aortic valve implantation (TAVI).

**Pre-TAVI Measurements**						
	**aPWV (m/s)**		**AIx@75 (%)**		**β_area_**	
	**R**	***p*-Value**	**R**	***p*-Value**	**R**	***p*-Value**
CS (%) ^1^	−0.40	0.016	0.11	0.508	−0.71	<0.001
CSR (1/s) ^1^	−0.23	0.184	0.13	0.458	−0.67	<0.001
**Post-TAVI Measurements**						
	**aPWV (m/s)**		**AIx@75 (%)**		**β_area_**	
	**R**	***p*-Value**	**R**	**R**	***p*-Value**	**R**
CS (%) ^1^	−0.09	0.583	0.08	0.632	−0.80	<0.001
CSR (1/s) ^1^	−0.11	0.528	0.09	0.573	−0.69	<0.001
**Change between Pre- and Post-TAVI Measurements**						
	**ΔaPWV (m/s)**		**ΔAIx@75 (%)**		**Δβ_area_**	
	**R**	***p*-Value**	**R**	***p*-Value**	**R**	***p*-Value**
ΔCS (%) ^2^	−0.34	0.055	0.08	0.675	−0.64	<0.001
ΔCSR (1/s) ^2^	−0.21	0.242	−0.02	0.857	−0.34	<0.001

^1^ Spearman correlation coefficient; ^2^ Pearson correlation coefficient.

## Data Availability

The data that supports the results are available upon request from the corresponding author (Leonie Arnold: leonie.arnold@med.uni-muenchen.de).

## Supplementary Materials

The following supporting information can be downloaded at: https://www.mdpi.com/article/10.3390/jcm13010222/s1, Table S1: Pearson correlation matrix of stiffness indices circumferential strain (CS, %), strain rate (CSR, 1/s), normalized stiffness index β_area_, arterial pulse wave velocity (aPWV, m/s), augmentation index normalized to heart rate (Alx@75, %) and patient characteristics, hemodynamic parameters, and laboratory parameters in patients with aortic stenosis (AS) undergoing transcatheter aortic valve implantation (TAVI); Table S2: Mixed linear regression model with a random intercept for Patient ID and arterial stiffness markers as outcomes variables for patients before and after transcatheter aortic valve transplantation (TAVI); Table S3: Patients with atrial fibrillation (paroxsysmal and permanent) versus patients without atrial fibrillation arterial stiffness markers before transcatheter aortic valve transplantation (TAVI).
