# Supplementary material for: Short-Term Changes in Arterial Stiffness Measured by 2D Speckle Tracking in Patients Undergoing Transcatheter Aortic Valve Implantation
Source: J Clin Med. 2023 Dec 30;13(1):222. doi: 10.3390/jcm13010222 (PMC10779940; doi:10.3390/jcm13010222)
Supplement: Supplementary file 1 [file jcm-13-00222-s001.zip › jcm-2728601-supplementary.pdf]

Supplementary materials

Table S1: Pearson correlation matrix of stiffness indices circumferential strain (CS), strain rate (CSR), normalized stiffness index $β_{area}$, arterial pulse wave velocity (aPWV), augmentation index normalized to heart rate (AIx@75) and patient characteristics, hemodynamic parameters and laboratory parameters in patients with aortic stenosis (AS) undergoing transcatheter aortic valve implantation (TAVI).

	Pre-CS (%)	Post-CS (%)	ΔCS (%)	Pre-CSR (1/s)	Post-CSR (1/s)	ΔCSR (1/s)	Pre-$β_{area}$	Post-$β_{area}$	Δ$β_{area}$
**Age (years)**	-0.32*	-0.10	0.29	-0.25	-0.11	0.22	0.25	0.23	-0.37*
**BMI (kg/m2)**	0.03	-0.23	-0.32*	0.25	-0.19	-0.34*	-0.05	0.05	0.16
**AVA (mm^2)[a]**	0.14	-0.15	-0.32*	0.06	-0.10	-0.24	-0.07	-0.08	0.07
**Pre-HR (bpm)**	-0.44*	-0.20	0.20	-0.13	-0.11	-0.01	0.21	-0.03	-0.37*
**Post-HR (bpm)**	-0.38*	-0.32*	0.019	0.02	-0.15	-0.12	0.22	0.02	-0.18
**ΔHR (bpm)**	0.19	-0.06	-0.25	0.16	-0.05	-0.14	-0.01	0.03	0.28
**Pre-MAP (mmHg)**	0.08	0.21	0.075	0.18	0.16	0.04	-0.01	-0.09	-0.10
**Post-MAP (mmHg)**	0.26	0.11	-0.33*	0.24	0.04	-0.22	-0.23	-0.24	0.03
**ΔMAP (mmHg)**	0.11	-0.15	-0.31*	0.02	-0.17	-0.20	-0.13	-0.01	0.10
**Pre-PVel (m/sec)**	0.01	0.21	0.13	0.09	0.19	0.24	0.07	-0.01	-0.04
**Post-PVel (m/sec)**	0.05	-0.08	-0.15	0.20	-0.04	-0.13	-0.11	-0.07	0.09
**ΔPVel (m/sec)**	0.01	-0.29	-0.21	-0.03	-0.26	-0.33*	-0.12	0.01	0.10
**Pre-MaxPG (mmHg)**	-0.03	0.10	0.12	0.03	0.02	0.20	0.10	0.11	-0.06
**Post-MaxPG (mmHg)**	0.10	-0.11	-0.32*	0.20	-0.09	-0.26	-0.11	-0.10	0.16
**ΔMaxPG (mmHg)**	0.06	-0.20	-0.25	-0.03	-0.15	-0.31*	-0.13	-0.12	0.13
**Pre-MPG (mmHg)**	0.01	0.24	0.14	0.12	0.23	0.24	0.06	-0.10	-0.10
**Post-MPG (mmHg)**	0.16	-0.06	-0.31	0.24	-0.03	-0.22	-0.09	-0.08	0.03
**ΔMPG (mmHg)**	0.02	-0.32	-0.24	-0.10	-0.32*	-0.34*	-0.05	0.12	0.04
**Pre-Cardiac index (l/min*1/m^2)[a]**	-0.06	0.11	0.16	-0.01	0.17	0.14	0.05	-0.15	-0.39*

**Post-Cardiac index (l/min*1/m²)**[a]	-0.02	0.19	0.10	0.02	0.12	0.14	-0.07	-0.14	-0.10
**Δ-Cardiac index (l/min*1/m²)**	0.11	0.03	-0.16	0.10	-0.06	-0.12	-0.12	0.10	0.37*
**Pre-Total vascular resistance (dyn·s/cm⁵)** [a]	0.14	0.15	-0.01	0.06	0.09	-0.01	-0.11	-0.04	-0.02
**Post-Total vascular resistance (dyn·s/cm⁵)** [a]	0.19	0.21	-0.04	0.24	0.26	0.05	-0.12	-0.25	-0.04
**ΔTotal vascular resistance (dyn·s/cm⁵)**	-0.01	-0.07	-0.01	0.09	0.04	-0.02	0.02	-0.06	0.08
**NT-proBNP (pg/mL)**[a]	-0.24	0.06	0.37*	-0.29	0.02	0.31*	0.16	0.02	-0.38*
**Total cholesterol (mg/dL)**[a]	0.19	0.19	0.09	0.17	0.15	0.02	-0.03	-0.16	-0.15
**Triglycerides (mg/dL)**[a]	-0.01	0.07	0.16	-0.04	0.09	0.09	0.02	-0.13	-0.14
**LDL-Cholesterol (mg/dL)**[a]	0.13	0.13	0.08	0.066	0.07	0.03	-0.01	-0.16	-0.17
**HDL-Cholesterol (mg/dL)**[a]	0.16	-0.03	-0.26	0.18	-0.06	-0.19	-0.07	-0.10	0.02
**Non-HDL-Cholesterol (mg/dL)**[a]	0.16	0.19	0.15	0.10	0.13	0.06	-0.01	-0.15	-0.19
**Sex**[b]	0.16	0.21	0.10	0.18	0.18	-0.06	0.02	-0.17	-0.09
**Diabetes**[b]	-0.01	0.14	0.09	0.11	0.20	0.23	-0.23	-0.02	0.18

[a]Spearman correlation coefficient

[b]Point-biserial correlation coefficient

*$p < 0.05$

Table S1 continued

	Pre-aPWV (m/s)	Post-aPWV (m/s)	ΔaPWV (m/s)	Pre-AIx@75 (%)	Post-AIx@75 (%)	ΔAIx@75 (%)
**Age (years)**	0.89*	0.90*	-0.20	0.04	-0.04	-0.17
**BMI (kg/m2)**	-0.19	-0.15	0.16	0.10	0.03	-0.05
**AVA (mm^2)[a]**	-0.18	-0.21	0.02	-0.02	0.05	0.10
**Pre-HR (bpm)**	-0.06	-0.05	0.07	0.04	0.19	0.14
**Post-HR (bpm)**	-0.26	-0.32*	0.04	-0.23	0.28	0.36*
**ΔHR (bpm)**	-0.24	-0.29	-0.04	-0.28	0.02	0.20
**Pre-MAP (mmHg)**	0.29	-0.07	-0.62*	0.15	-0.12	-0.25
**Post-MAP (mmHg)**	-0.12	0.19	0.49*	0.10	0.14	-0.08
**ΔMAP (mmHg)**	-0.30	0.27	0.90*	0.03	0.25	0.15
**Pre-PVel (m/sec)**	0.17	0.03	-0.04	0.28	-0.18	-0.33
**Post-PVel (m/sec)**	-0.04	-0.02	0.26	-0.03	-0.02	0.04
**ΔPVel (m/sec)**	-0.17	0.02	0.2	-0.25	0.03	0.27
**Pre-MaxPG (mmHg)**	0.14	-0.02	-0.13	0.24	-0.23	-0.39*
**Post-MaxPG (mmHg)**	-0.11	-0.01	0.36*	0.02	0.05	0.04
**ΔMaxPG (mmHg)**	-0.18	0.01	0.28	-0.23	0.14	0.36*
**Pre-MPG (mmHg)**	0.15	0.04	-0.09	0.24	-0.14	-0.34*
**Post-MPG (mmHg)**	-0.01	0.02	0.26	0.08	-0.04	-0.01
**ΔMPG (mmHg)**	-0.21	-0.10	0.08	-0.29	0.08	0.40*
**Pre-Cardiac index (l/min*1/m^2)[a]**	0.04	-0.04	-0.25	-0.36*	0.28	0.47*
**Post-Cardiac index (l/min*1/m^2)[a]**	-0.20	-0.10	0.12	0.01	-0.09	-0.14
**Δ-Cardiac index (l/min*1/m^2)**	-0.20	-0.09	0.36	0.44*	-0.38*	-0.59*
**Pre-Total vascular resistance (dyn·s/cm^5) [a]**	0.11	0.03	-0.20	0.57*	-0.17	-0.57*

**Post-Total vascular resistance (dyn·s/cm^5) [a]**	-0.20	-0.06	0.07	0.05	0.51*	0.32
**ΔTotal vascular resistance (dyn·s/cm^5)**	-0.17	0.01	0.25	-0.37*	0.48*	0.70*
**NT-proBNP (pg/mL)[a]**	0.43*	0.50*	0.09	-0.13	-0.02	0.10
**Total cholesterol (mg/dL)[a]**	0.06	-0.01	-0.09	0.33	0.15	-0.09
**Triglycerides (mg/dL)[a]**	-0.10	-0.01	-0.01	0.17	0.03	0.09
**LDL-Cholesterol (mg/dL)[a]**	0.18	0.05	-0.15	0.26	-0.01	-0.17
**HDL-Cholesterol (mg/dL)[a]**	-0.18	-0.20	0.20	0.14	0.19	-0.09
**Non-HDL-Cholesterol (mg/dL)[a]**	0.21	0.08	-0.23	0.27	0.04	-0.10
**Sex[b]**	-0.21	-0.25	-0.07	0.22	0.52*	0.24
**Diabetes[b]**	-0.15	-0.06	0.01	-0.07	-0.01	0.03

[a]Spearman correlation coefficient

[b]Point-biserial correlation coefficient

*$p < 0.05$

Table S2: Mixed linear regression model with a random intercept for Patient ID and arterial stiffness markers as outcomes variables for patients before and after aortic valve transplantation (TAVI).

	Circumferential strain (%)			Circumferential strain rate (1/s)			Normalized stiffness index β_{area}			Arterial Pulse wave velocity (m/s)			Augmentation index normalized to heart rate AIx@75 (%)		
Predictors	Est.	CI	p	Est.	CI	p	Est.	CI	p	Est.	CI	p	Est.	CI	p
(Intercept)	18.65	10.48; 26.83	**<0.001**	1.45	-0.96; 3.85	0.234	-2.81	-13.16; 7.53	0.590	-8.81	-10.03; -7.58	**<0.001**	-37.35	-76.55; 1.84	0.061
Timepoint	0.84	0.20; 1.47	**0.011**	0.33	0.14; 0.52	**0.001**	-0.45	-1.07; 0.17	0.149	-0.01	-0.11; 0.10	0.894	-6.96	-11.24; -2.69	**0.002**
Age (yrs)	-0.10	-0.18; -0.02	**0.017**	-0.00	-0.03; 0.02	0.693	0.07	-0.04; 0.18	0.210	0.21	0.19; 0.22	**<0.001**	0.29	-0.06; 0.64	0.101
Sex (female)	1.26	0.10; 2.42	**0.033**	0.26	-0.08; 0.60	0.129	-0.11	-1.70; 1.49	0.895	0.08	-0.09; 0.25	0.342	10.49	5.31; 15.67	**<0.001**
HR (bpm)	-0.08	-0.11; -0.04	**<0.001**	-0.00	-0.01; 0.01	0.635	0.03	-0.01; 0.07	0.164	-0.00	-0.01; 0.00	0.321	0.12	-0.07; 0.31	0.203
MAP (mm Hg)	-0.01	-0.05; 0.02	0.419	0.00	-0.01; 0.01	0.819	0.01	-0.03; 0.04	0.701	0.05	0.04; 0.05	**<0.001**	0.30	0.11; 0.49	**0.003**
Random Effects															
σ^2	1.80			0.17			1.56			0.05			86.65		
τ_{00}	1.52 Pat_ID			0.12 Pat_ID			3.91 Pat_ID			0.03 Pat_ID			0.00 Pat_ID		
ICC	0.46			0.42			0.71			0.35			0.08		
N	44 Pat_ID			44 Pat_ID			44 Pat_ID			43 Pat_ID			43 Pat_ID		
Observations	84			84			84			79			79		

Marginal R^2 / Conditional R^2	0.274 / 0.606	0.116 / 0.488	0.051 / 0.729	0.961 / 0.975	0.328 / NA

Table S3: Patients with atrial fibrillation (paroxsysmal and permanent) versus patients without atrial fibrillation arterial stiffness markers before transcatheter aortic valve transplantation (TAVI).

Pre-TAVI		Patients with atrial fibrillation		Patients without atrial fibrillation	
Parameter	N	Median ± IQR	N	Median ± IQR	p-value[a]
**Stiffness indices**					
CS (%)	16	4.08 ± 2.008	28	4.69 ± 2.404	0.652
CSR (1/s)	16	0.83 ± 0.481	28	0.93 ± 0.570	0.826
β_{area}	16	5.27 ± 2.682	27	4.99 ± 2.381	0.418
aPWV (m/s)	17	12.10 ± 1.533	21	11.57 ± 1.367	0.106
Alx@75 (%)	17	25.67 ± 11.500	21	32.33 ± 14.667	0.229

[a] p-value calculated by Wilcoxon rank sum test

CS = Circumferential strain. CSR = Circumferential strain rate. β_{area} = Normalized stiffness index based on area. aPWV = Arterial pulse wave velocity. Alx@75 = Augmentation index normalized to heart rate
